# Progress in immunological research on innate effectors in xenotransplantation

**DOI:** 10.1007/s00595-026-03267-3

**Published:** 2026-03-19

**Authors:** Shuji Miyagawa, Akira Maeda, Chiyoshi Toyama, Shuhei Kogata, Yuki Noguchi, Rei Matsuura, Hiroshi Eguchi, Takehisa Ueno, Hiroomi Okuyama

**Affiliations:** Department of Pediatric Surgery, Osaka University Graduate School of Medicine, 2-2 Yamadaoka, Suita, 565-0871 Osaka Japan

**Keywords:** Xenotransplantation, Macrophages, Neutrophils, SP-A/D, CD177

## Abstract

In xenotransplantation research, the control of innate immune cells is problematic. In this review, we focus on molecules, which are control-related findings obtained from known immune responses, and investigate whether they can be applied to control macrophages and neutrophils. The “missing-self” is a well-known recognition system for natural killer cells, but it has been shown that human leukocyte antigen (HLA)-G1, rather than HLA- E, exerts this control effect on macrophages and neutrophils. Moreover, CD47 is known as a “don’t eat me signal,” and its action can be applied to the control not only of macrophages but also of neutrophils. However, this molecule is likely influenced strongly by thrombospondin-1. In contrast, when the pulmonary surfactant protein D is converted to a membrane-type molecule, it exerts a stronger regulatory effect on macrophages and neutrophils than CD47. Regarding “contact inhibition,” the suppression of cytotoxicity by CD31 was observed in neutrophils, but not well in macrophages. In contrast, CD177, a ligand with high affinity for CD31, showed a significant regulatory effect on macrophages and neutrophils through CD31 expression. These findings are important for the future production of genetically edited pigs.

## Introduction

### History of xenoresearch

**Fig. 1:**
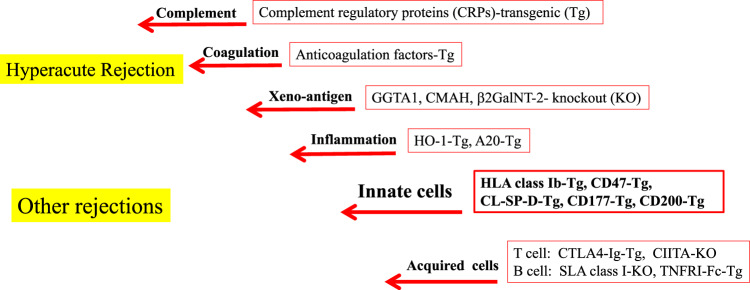
The progress of xenotransplantation research. The first breakthrough was the discovery that hyperacute rejection was caused by species differences in the complement system. Research into the coagulation system continued. Main species-specific carbohydrate antigens were identified, but it is now relatively easy to knock out these. Therefore, the control of innate immune cells is becoming increasingly important

Clinical xenotransplantation has been attempted many times over recent years. Basic immunological research on this topic began in earnest in the late 1980s. The mechanism of hyperacute rejection in xenotransplantation remains unclear; however, it has been discovered that species differences in the complement system are the main cause, and studies on complement regulatory factors have been actively conducted [1, 2]. Therefore, the first genetic modification in pigs focused on complement regulatory factors (CRPs), such as membrane cofactor protein (CD46), decay-accelerating factor (DAF: CD55) [3], and CD59. The first DAF transgenic (Tg) pigs were reported in 1994 [4], and these were followed by other CRP-Tg pigs [5]. Studies have progressed to species differences in coagulation factors (Fig. 1). Coagulation-related molecules, such as thrombomodulin, tissue factor pathway inhibitor (TFPI), endothelial cell protein C receptor, CD39, and CD73, have been studied [6].

The next focus was on xeno-glycosil antigens [7]. The existence of the α-galactosil epitope (α-gal) (alpha-1,3-galactosyltransferase: GGTA1) [8], the largest xenoantigen between pigs and humans, was revealed around the time of the third International Xenotransplantation Association (IXA). Initially, it was thought that knocking out the gene was impossible in pigs because knockout (KO) pig embryonic stem cells have not been established. Therefore, other methods to reduce the α-gal epitope [9], such as overexpression of α−1,2 fucosyltransferase (FT) [10], α−2,3/α−2,6 sialyltransferase (ST) [11, 12] and acetylglucosaminyltransferase-III (GnT-III) [13–16], were studied first [17, 18]. Fortunately, by combining gene modification technology with (fetal) fibroblast and nuclear transfer technology, α-gal-KO pigs were established in 2002 [19]. This was followed by the Hanganutziu-Deicher (H-D) antigen (cytidine monophospho-N- acetylneuraminic acid hydroxylase (CMAH))-KO pigs [20–22]. Recently, Sd^a^ (β4GalNT2) has been knocked out, but this glycan is a blood-group antigen [23, 24]. Furthermore, studies have been conducted on anti-apoptotic and anti-inflammatory genes, such as heme oxygenase 1 [25] and A20 [26]. Specie differences in the expression of these genes have not been studied extensively. In genetically edited pigs, they are also thought to regulate T and B cells. Class II trans-activator dominant-negative (CIITA-DN)-Tg [27, 28], CIITA-KO, Swine leukocyte antigen (SLA) class I-KO [29], FasL-Tg, CTLA4-Ig-Tg, and tumor necrosis factor receptor I IgG-Fc (TNFRI-Fc)-Tg [30], have been proposed.

With the improvement of genetic recombination technology, many types of CRPs, anticoagulants, and carbohydrate antigens have been selected as targets. Pigs, such as 10 genetic edit (10GE: Revivicor, Inc.) [31] and 10 genetic modifications (10GM: eGenesis, Inc.) [32], have been created, and clinical applications are already underway [33, 34]. However, considering that spatial transcriptions and single-cell RNA sequencing analysis in pig kidney and heart transplants from brain-dead patients have revealed significant infiltration of cells of the innate immune system [35, 36], the rejection reaction by innate immune cells comprising natural killer (NK) cells [37], macrophages including dendric cells [38], and neutrophils [39, 40] could be the next obstacle in xenotransplantation. Therefore, to monitor the infiltration of these innate immune cells, single-cell RNA sequencing analysis of biopsy samples is considered effective; however, only CD47 [41] has been genetically introduced into 10GE and 10GM pigs.

While this review focuses on genetic engineering, attempts are also being made clinically to control individual rejection reactions using drugs. For example, the use of monkeys as transplant animals was once considered. In a 1964 clinical trial, a chimpanzee kidney survived for more than 6 months. The immunosuppressants used at that time were steroids, azathioprine, and ATG [42]. Currently, issues such as animal welfare and zoonotic diseases prohibit the use of monkeys as donors. On the other hand, since the focus has been on hyperacute rejection, before the development of transgenic pigs with CRPs, various drugs inhibiting complement activity were tested in preclinical studies (Table 1) [43–68]. Anticoagulants were also used. Since α-Gal was identified as a xeno-glyco antigen, drugs to suppress antibodies against α-Gal were developed until the establishment of knockout pigs (Table 1). Meanwhile, to address the three subjects discussed here, pigs gen-edited for 10GE and 11GE have been genetically modified to target HLA-E and CD47 genes for NK cells and macrophages, respectively. Unfortunately, few drugs have been developed to selectively control innate immune cells.

**Table 1:** Immunological drugs for anti-xeno-rejection

	Name	Contents	Ref.
Complement regulatory drugs			
	CVF	Cobra venom factor of the Indian cobra *Naja naja*.	[43]
	K-76COOH	K-76 monocarboxylic acid	[44]
	sCR1(TP-10)	Lacking the transmembrane and cytoplasmic region of CR1	[45]
	APT070 (Mirococept)	SCR1-3 of CR1 with a membrane-targeting amphiphilic peptide	[46]
	C1-INH	C1 esterase inhibitor	[47]
	Pexelizumab	A single chain version of the anti-C5 humanized mAb.	[48]
	Eculizumab	An anti-C5 humanized mAb.	[49]
	Complement activation blocker-2 (CAB-2)	A chimeric protein derived from human DAF and MCP.	[50]
	Compstatin	A C3-binding peptide (ICVVQDWGHHRCT-NH2)	[51]
	Ac-PepA	An acetylated form of Pep A: (ASGAPAPGPAGPLRPMF)	[52]
Additional new drugs for complement regulation			
	Crovalimab	Anti-C5 Inhibitors	[53]
	Pegcetacoplan	Anti-C3 Inhibitors	[54]
	Iptacopan	Anti-Complement Factor B Inhibitors	[55]
	Bemilkopan	Anti-Complement Factor D Inhibitors	[56]
Anti-coagulation drugs			
	Heparin		-
	FUT-175	Nafamstat mesilate	[44]
	C1-INH	C1 esterase inhibitor	[47]
Anti-Gal drugs			
	GAS914	A soluble Gal(⍺1-3)Gal polymer	[57]
	TRFA	Anti-Gal-polyethylenglycol conjugate	[58]
Anti-inflamatory			
	Tocilizumab	Anti-IL-6 Receptor	[59]
	Anti-TNF-α		[60]
Anti-macrophage drugs			
	LC	Liposome Clodronate	[61]
	PQA-18	Prenylated quinolinecarboxylic acid compound-18	[62]
Anti-second signal & anti-T, B drugs			
	CTLA4-Ig		[63]
	aglycosyl hu5c8	Anti-CD154 mAb	[64]
	TNX-1500	Anti-CD154 mAb	[65]
	AT-1501	Anti-CD154 mAb	[66]
	Anti-CD40 mAb		[67]
	Anti-CD38 mAb		[68]

Meanwhile, kidney transplants using these gene-edited pigs have been performed since 2024, based on the results of long-term preclinical trials using monkeys. Although the first clinical case at Harvard University did not survive long because of heart disease [34, 69, 70], the second clinical kidney transplant survived for 9 months. However, gene-edited pigs are still in their early stages, and further modifications are necessary. Furthermore, the control of innate immune cells is thought to be essential for stable engraftment in porcine xenotransplants, including those of the heart, lung, liver, and islet cells. This study focuses on the similarities between macrophages and neutrophils, among other innate immune cells, and outlines molecular biological approaches to their control from three perspectives.

### Missing-self

It is well established that NK cell activity is strongly related to xenograft rejection, and methods for controlling this activity have been investigated using two approaches [37]. One approach is from the perspective of glycobiology [71–73]. It has been verified that reducing α-gal epitopes on pig cells reduces the aggressiveness of NK cells against pig cells. This is because NK cells recognize α-gal epitopes via CD161 [74, 75]. Moreover, FcγRIIC and FcγRIIIA on NK cells react with antibodies, while DAF (CD55) is associated with NK cells, which may control its movement [76]. A second approach is to use major histocompatibility complex (MHC) class Ib (HLA-G [77, 78] and HLA-E [79, 80]). NK cells are inherently aggressive cells, and their aggressiveness is determined by the balance of signals from aggressive receptors with immunoreceptor tyrosine-based activation motifs (ITAMs) on their cell surface (for example, CD158d for HLA-G and CD94/NKG2C for HLA-E) and inhibitory receptors with immunoreceptor tyrosine-based inhibitory motifs (ITIMs) (e.g., ILT-2 [CD85j] and ILT-4 [CD85d] for HLA-G and CD94/NKG2A for HLA-E) [81–83].

Normally, NK cells receive inhibitory signals from the HLA class I molecules that are present in most self-cells in the body. In xenotransplantation, because the target pig cells do not express human class I molecules, the inhibitory receptors on NK cells cannot transmit signals, and then begin attacking. To prevent human NK cells from attacking pig cells, a study has been conducted to forcibly express human MHC class I molecules and suppress the xeno-cytotoxic activity by NK cells. HLA class I consists of classes Ia and Ib. Class Ia comprises A, B, and C, each of which has a large number of alleles and even if one of them is expressed in a pig, it cannot control the NK cells of the recipient with the other alleles. It is thought that this will increase the antigen levels. Therefore, in xenotransplantation, HLA-G1 and HLA-E from class Ib, which have few alleles, are used for gene transfer in pigs.

The “missing self” theory is known as the foreign body recognition system of NK cells. In this review, we investigated whether this system is not limited to NK cells but also applies to macrophages and neutrophils. To give a brief overview of the macrophage activation signals outline, macrophages are activated by stimulation from Fc-receptors that react to antibodies, C-receptors that correspond to each complement factor (CR3, CR4, and C1q-receptor) [84], and scavenger receptors that react to denatured low-density lipoprotein. Macrophages also have toll-like receptors (TLRs) that are activated by recognizing damage-associated molecular patterns (DAMPs), such as high mobility group box-1 (HMGB1). The Mincle ligand, which recognizes β-glucosylceramide on dead cells as the “eat me signal”, is also expressed [85].” Furthermore, when phosphatidylserine is exposed on the cell surface as a result of apoptosis caused by ischemia/reperfusion (I/R) injury, T-cell immunoglobulin and mucin domain 4 (Tim-4) [86] and milk fat globule-epidermal growth factor 8 (MFG-E8) [87] on the surface of macrophages recognize this and activate the signals. To suppress these signals, we examined the expression of ILT-2 and ILT- 4 in macrophages, which are ligands for HLA-G1 [82]. ILT-2 was stably expressed from the monocytes stage, while ILT-4 expression increased during maturation. In contrast, NKG2A, a ligand of HLA-E, was stably expressed in mature macrophages [81]. As a result, it was confirmed that expressing HLA-G1 or HLA-E in pig cells could prevent macrophage attacks.

Next, we investigated whether neutrophils also perform this function. Neutrophils, originally known as “microphages”, are cells of the innate immune system, specialized in attacking invading foreign substances [88]. The aggressiveness of neutrophils [89], like that of macrophages, is composed of two main factors, Fc receptors such as FcγRIIA-C and FcγRIIIB, and the complement receptors, CR1, CR3 and CR4. Moreover, neutrophils adhere to swine endothelial cells (SECs) via CD82 [90], a membrane-like tetraspanin. Neutrophils also express TLR, Mincle, and receptor for advanced glycation end-product, which are related to neutrophil activation. Therefore, in xenotransplantation, it is essential to control neutrophils, and a study is underway to explore ways to achieve this. The expression of ILT-2, ILT-4, and NKG2A was examined in leukocytes. The expression of ILT-2 and NKG2A was insufficient, but the expression of ILT-4 was relatively high at 60%–70%. For this reason, a cytotoxicity test of neutrophils against SEC showed that when HLA-G1 molecules were expressed, they controlled the reaction significantly and suppressed the associated reactive oxygen species (ROS) and netosis reactions. In contrast, there was no difference in the degree of suppression of neutrophil attack by HLA-E [91].

### “Don’t eat me” signal

Until recently, the only widely known method for controlling macrophages was to express CD47 [37] on pig cells and bind this to signal regulatory protein α (SIRPα) on the surface of macrophages, to act as a “don’t eat me” signal. This molecule may also have significant species specificity, even between human and non-human primates [92]. This signal was first reported in 2000 [93] and has since been applied in the field of xenotransplantation. Pigs expressing hCD47 have been created. This molecule binds to SIRPα, which exists stably on the membrane of macrophages, and the ITIM present in the cells is activated, exerting an inhibitory effect. There are four other molecules in SIRP besides α, β1 and β2 have ITAM, but do not react with CD47, γ reacts with CD47, but is much weaker than α in the affinity, and δ is a secretory type [94]. CD47 also reacts with thrombospondin-1 (TSP-1) and other molecules, such as SHPS-1 and αVβ3, resulting in a relatively weak interaction with SIRPα. At the same time, there are concerns about transgenic human CD47 expression in pig renal podocytes, and constitutive stimulation by serum human TSP-1 may lead to kidney graft damage [95, 96]. Thus, TSP-1 is a secretory molecule released by vascular endothelial cells, epithelial cells, fibroblasts, and other cell types, which binds to many ligands, including CD47, CD36, and tissue factory pathway inhibitor (TFPI). TSP-1 inhibits CD47-SIRPα signaling and promotes phagocytosis by macrophages, while also contributing to platelet aggregation [97]. Consequently, its secretion increases in ischemic tissues and damaged grafts, potentially leading to graft rejection [98, 99]. Conversely, the N-terminal collagen domains of pulmonary surfactants SP-A and SP-D induce inflammation by binding to the calreticulin/CD91 receptor complex, but their carbohydrate-recognition domains (CRDs) induce inhibitory signals to macrophages by binding to SIRPα [100]. Regarding the control of macrophages, it has been reported that membrane- type SP-A/D (CL-SP-A/D) [101–103], which avoids the collagen domain that causes inflammation in SP-A and SP-D and replaces only the CRD portion that binds to SIRPα with another molecule, human collectin placenta 1 (CL-P1) [104], is effective in suppressing macrophages. Furthermore, CD47 and CL-SP-D have different binding sites for the SIRPα molecule. CD47 binds to the D1 portion of SIRPα, while the CRD portion of SP-D binds to the sialic acid in the D2-D3 portion [105, 106]. These molecules are expected to exhibit both synergistic and additive effects.

We also studied CD200 [107] and TIGIT [108] and verified their control functions against macrophages in SECs. We reported that changes in carbohydrate antigens on SECs, such as overexpression of α2,6 sialic acid, are also effective in controlling macrophages [109–111]. Moreover, the question remains about whether this “don’t eat me” signal acts not only on macrophages but also on neutrophils. When the inhibitory effects of these molecules on neutrophils were examined in vitro, both molecules were found to be effective; however, CL-SP-D was more effective than CD47 in suppressing the cytotoxic effect, the ROS response, and the netosis response [112].

## Contact inhibition

CD31 is an adhesion factor that exerts contact inhibition function between CD31 molecules and contains ITIMs in the cell [113, 114]. It has been investigated whether this molecule’s cytostatic function can inhibit macrophage and neutrophil attack on porcine cells. CD31, commonly known as the platelet endothelial cell adhesion molecule, is expressed on many cells, including endothelial cells, platelets, granulocytes, macrophages, and dendritic cells. No mutual reaction was observed between human and porcine CD31. On the other hand, CD177 uses CD31 as a counter-receptor. This molecule is expressed in almost half of the neutrophils and is involved in the interaction between neutrophils and endothelial cells [115]. Importantly, the binding affinity between CD177 and CD31 is much stronger than that between CD31 and CD31.

Human macrophages express CD31, but they do not interact with pig CD31 on SECs. We investigated whether the expression of human CD31 on SECs could inhibit a human macrophage attack on SECs via CD31 on macrophages. However, a cytotoxicity assay of macrophages against SECs did not reveal a significant inhibitory effect via CD31 interaction [116]. Next, CD177 molecules were expressed on SECs and the inhibitory effect was examined in a cytotoxicity test against the SECs of macrophages. A significant inhibitory effect was found [117], which may be attributable to the stronger affinity between CD31 and CD177 than between CD31 and CD31 [115].

Next, CD31 and CD177 were expressed on SECs to examine their inhibitory effect on a neutrophil attack. First, the expression of CD31 in neutrophils was confirmed, so when CD31 and CD177 were expressed on SECs, the attack on SECs by neutrophils was significantly suppressed by CD31 [118] and CD171 [119] on SECs. Therefore, it is difficult to avoid attacks from both macrophages and neutrophils by using the function of the adhesion molecule CD31, but we think it is possible to avoid simultaneous attacks from both by using one of its ligands, CD177. Moreover, it has been reported that CD177 tends to suppress neutrophil-mediated SEC attack more effectively than CD31 [119].

## Conclusion

The present study focused on whether insights gained from known immune responses, such as “don’t eat me” signals, missing-self, and adhesion molecules, could help control xenograft rejection and pave the way for future xenotransplantation success. We examined the common control methods for macrophages and neutrophils, which have similar cellular origins. Xenotransplantation studies began with the complement system and xeno-glycosil antigens, and 10GE and 10GM have been used in clinical trials. However, because of the importance of innate immune cells in xenotransplantation, only CD47 was genetically transferred, and the molecules introduced in them did not include HLA-Ib, which is known to control NK cells. Thus, considering the importance of controlling macrophages and neutrophils, in vitro data suggest that it is necessary to express genes such as membrane- type SP-D, HLA-Ib (E and G1), and CD177 (Table 2) [120].

**Table 2:** Key molecules in the regulation of innate immune cells for xenotransplantation

Molecule	Ligand	Monocyte/macrophage	Neutrophil	Ref.
CD47	SIRPα	+	+	[37],[79]
SP-A/D	SIRPα	++	++	[69],[70],[79]
HLA-E	NKG2A	+	?	[48],[58]
HLA-G1	ILT-2/ILT-4	+	+	[49],[58]
CD31	CD31	?	+	[83],[85]
CD177	CD31	+	+	[84],[86]

## References

[1] Miyagawa S, Hirose H, Shirakura R, Naka Y, Nakata S, Kawashima Y, et al. The mechanism of discordant xenograft rejection. Transplantation. 1988;46:825–30. 3061076 10.1097/00007890-198812000-00007

[2] Miyagawa S, Yamamoto A, Matsunami K, Wang D, Takama Y, Ueno T, et al. Complement regulation in the GalT KO era. Xenotransplantation. 2010;17:11–25. 20149185 10.1111/j.1399-3089.2010.00569.x

[3] Dalmasso AP, Vercellotti GM, Platt JL, Bach FH. Inhibition of complement-mediated endothelial cell cytotoxicity by decay-accelerating factor. Potential for prevention of xenograft hyperacute rejection. Transplantation. 1991;52:530–3. 1716798 10.1097/00007890-199109000-00029

[4] Cozzi E, White DJ. The generation of transgenic pigs as potential organ donors for humans. Nat Med. 1995;1:964–6. 7585226 10.1038/nm0995-964

[5] Loveland BE, Milland J, Kyriakou P, Thorley BR, Christiansen D, Lanteri MB, et al. Characterization of a CD46 transgenic pig and protection of transgenic kidneys against hyperacute rejection in non-immunosuppressed baboons. Xenotransplantation. 2004;11:171–83. 14962279 10.1046/j.1399-3089.2003.00103.x

[6] Cowan PJ. Coagulation and the xenograft endothelium. Xenotransplantation. 2007;14:7–12. 17214700 10.1111/j.1399-3089.2006.00368.x

[7] Miyagawa S, Maeda A, Kawamura T, Ueno T, Usui N, Kondo S, et al. A comparison of the main structures of N-glycans of porcine islets with those from humans. Glycobiology. 2014;24:125–38. 24100142 10.1093/glycob/cwt088

[8] Galili U, Swanson K. Gene sequences suggest inactivation of alpha-1,3-galactosyltransferase in catarrhines after the divergence of apes from monkeys. Proc Natl Acad Sci U S A. 1991;88:7401–4. 1908095 10.1073/pnas.88.16.7401 PMC52303

[9] Cooper DKC. Depletion of natural antibodies in non-human primates - a step towards successful discordant xenografting in humans. Clin Transplant. 1992;6:178–83. 10147930

[10] Sandrin MS, Fodor WL, Mouhtouris E, Osman N, Cohney S, Rollins SA, et al. Enzymatic remodelling of the carbohydrate surface of a xenogenic cell substantially reduces human antibody binding and complement-mediated cytolysis. Nat Med. 1995;1:1261–7. 7489406 10.1038/nm1295-1261

[11] Tanemura M, Miyagawa S, Koyota S, Koma M, Matsuda H, Tsuji S, et al. Reduction of the major swine xenoantigen, the alpha-galactosyl epitope by transfection of the alpha2,3-sialyltransferase gene. J Biol Chem. 1998;273:16421–5. 9632707 10.1074/jbc.273.26.16421

[12] Koma M, Miyagawa S, Honke K, Ikeda Y, Koyota S, Miyoshi S, et al. Reduction of the major xenoantigen on glycosphingolipids of swine endothelial cells by various glycosyltransferases. Glycobiology. 2000;10:745–51. 10910978 10.1093/glycob/10.7.745

[13] Tanemura M, Miyagawa S, Ihara Y, Matsuda H, Shirakura R, Taniguchi N. Significant downregulation of the major swine xenoantigen by N-acetylglucosaminyltransferase III gene transfection. Biochem Biophys Res Commun. 1997;235:359–64. 9199197 10.1006/bbrc.1997.6784

[14] Miyagawa S, Murakami H, Takahagi Y, Nakai R, Yamada M, Murase A, et al. Remodeling of the major pig xenoantigen by N-acetylglucosaminyltransferase III in transgenic pig. J Biol Chem. 2001;276:39310–9. 11486004 10.1074/jbc.M104359200

[15] Takahagi Y, Fujimura T, Miyagawa S, Nagashima H, Shigehisa T, Shirakura R, et al. Production of alpha 1,3-galactosyltransferase gene knockout pigs expressing both human decay-accelerating factor and N-acetylglucosaminyltransferase III. Mol Reprod Dev. 2005;71:331–8. 15806557 10.1002/mrd.20305

[16] Komoda H, Miyagawa S, Omori T, Takahagi Y, Murakami H, Shigehisa T, et al. Survival of adult islet grafts from transgenic pigs with N-acetylglucosaminyltransferase-III (GnT-III) in cynomolgus monkeys. Xenotransplantation. 2005;12:209–16. 15807771 10.1111/j.1399-3089.2005.00206.x

[17] Ogawa H, Muramatsu H, Kobayashi T, Morozumi K, Yokoyama I, Kurosawa N, et al. Molecular cloning of endo-beta -galactosidase C and its application in removing alpha -galactosyl xenoantigen from blood vessels in the pig kidney. J Biol Chem. 2000;275:19368–74. 10858461 10.1074/jbc.M001888200

[18] Miyagawa S. Xenotransplantation and glycomedicine. Comprehensive Glycoscience. Johannis P Kamerling, editors ELSEVIER Press. 2007;533 – 53.

[19] Dai Y, Vaught TD, Boone J, Chen SH, Phelps CJ, Ball S, et al. Targeted disruption of the alpha1,3-galactosyltransferase gene in cloned pigs. Nat Biotechnol. 2002;20:251–5. 11875425 10.1038/nbt0302-251

[20] Irie A, Koyama S, Kozutsumi Y, Kawasaki T, Suzuki A. The molecular basis for the absence of N-glycolylneuraminic acid in humans. J Biol Chem. 1998;273:15866–71. 9624188 10.1074/jbc.273.25.15866

[21] Lutz AJ, Li P, Estrada JL, Sidner RA, Chihara RK, Downey SM. Double knockout pigs deficient in N-glycolylneuraminic acid and galactose alpha-1,3-galactose reduce the humoral barrier to xenotransplantation. Xenotransplantation. 2013;20:27–35. 23384142 10.1111/xen.12019

[22] Miyagawa S, Matsunari H, Watanabe M, Nakano K, Umeyama K, Sakai R, et al. Generation of α1,3-galactosyltransferase and cytidine monophospho-N-acetylneuraminic acid hydroxylase gene double-knockout pigs. J Reprod Dev. 2015;61:449–57. 26227017 10.1262/jrd.2015-058 PMC4623151

[23] Byrne GW, Du Z, Stalboerger P, Kogelberg H, McGregor CGA. Cloning and expression of porcine β1,4 N-acetylgalactosaminyl transferase encoding a new xenoreactive antigen. Xenotransplantation. 2014;21:543–54. 25176027 10.1111/xen.12124 PMC4262693

[24] Lo PC, Eguchi H, Sakai R, Maeda A, Kogata S, Toyama C et al. Reaction to the porcine cells with or without b4GalNT2. Transplant Proc. 2020;52:1916-8. 10.1016/j.transproceed.2020.01.154 32482451

[25] Petersen B, Ramackers W, Lucas-Hahn A, Lemme E, Hassel P, Queisser AL, et al. Transgenic expression of human heme oxygenase-1 in pigs confers resistance against xenograft rejection during ex vivo perfusion of porcine kidneys. Xenotransplantation. 2011;18:355–68. 22168142 10.1111/j.1399-3089.2011.00674.x

[26] Oropeza M, Petersen B, Carnwath JW, Lucas-Hahn A, Lemme E, Hassel P, et al. Transgenic expression of the human A20 gene in cloned pigs provides protection against apoptotic and inflammatory stimuli. Xenotransplantation. 2009;16:522–34. 20042052 10.1111/j.1399-3089.2009.00556.x

[27] Yun S, Gustafsson K, Fabre JW. Suppression of human anti-porcine T-cell immune responses by major histocompatibility complex class II transactivator constructs lacking the amino terminal domain. Transplantation. 1998;66:103–11. 9679829 10.1097/00007890-199807150-00016

[28] Hara H, Witt W, Crossley T, Long C, Isse K, Fan L, et al. <article-title update=“added”> Human dominant‐negative class <scp>II</scp> transactivator transgenic pigs – effect on the human anti‐pig <scp>T</scp> ‐cell immune response and immune status. Immunology. 2013;140:39–46. 23566228 10.1111/imm.12107 PMC3809704

[29] Reyes LM, Estrada JL, Wang ZY, Blosser RJ, Smith RF, Sidner RA, et al. <article-title update=“added”>Creating class I MHC–null pigs using guide RNA and the Cas9 endonuclease. J Immunol. 2014;193:5751–7. 25339675 10.4049/jimmunol.1402059 PMC5922270

[30] Park SJ, Cho B, Koo OJ, Koo OJ, Kim H, Kang JT, et al. Production and characterization of soluble human TNFRI-Fc and human HO-1(HMOX1) transgenic pigs by using the F2A peptide. Transgenic Res. 2014;23:407–19. 24497084 10.1007/s11248-013-9780-x

[31] Mohiuddin MM, Singh AK, Scobie L, Goerlich CE, Grazioli A, Saharia K, et al. Graft dysfunction in compassionate use of genetically engineered pig-to-human cardiac xenotransplantation: a case report. Lancet. 2023;402:397–410. 37393920 10.1016/S0140-6736(23)00775-4 PMC10552929

[32] Kawai T, Williams WW, Elias N, Fishman JA, Crisalli K, Longchamp A, et al. Xenotransplantation of a Porcine Kidney for End-Stage Kidney Disease. N Engl J Med. 2025;392:1933–40. 39927618 10.1056/NEJMoa2412747

[33] Griffith BP, Goerlich CE, Singh AK, Rothblatt M, Lau CL, Shah A, et al. Genetically modified porcine-to-human cardiac xenotransplantation. N Engl J Med. 2022;387:35–44. 35731912 10.1056/NEJMoa2201422 PMC10361070

[34] Hirose T, Hotta K, Kawai T. Current progress in kidney xenotransplantation: time to proceed to clinical trials. Int J Urol. 2025;0:1–10. 10.1111/iju.70173 40631714

[35] Cheung MD, Asiimwe R, Erman EN, Fucile CF, Liu S, Sun CW, et al. Spatiotemporal immune atlas of a clinical-grade gene-edited pig-to-human kidney xenotransplant. Nat Commun. 2024;15:3140. 38605083 10.1038/s41467-024-47454-7 PMC11009229

[36] Giarraputo A, Morgand E, Stern J, Mezine F, Coutance G, Goutaudier V, et al. Characterizing the immune response in pig-to-human heart xenograftsusing a multimodal diagnostic system. Circulation. 2025. 10.1161/CIRCULATIONAHA.125.074971. 41036838 10.1161/CIRCULATIONAHA.125.074971 PMC12655869

[37] Puga Yung G, Schneider MKJ, Seebach JD. The role of NK cells in pig-to-human xenotransplantation. J Immunol Res. 2017;2017:4627384. 29410970 10.1155/2017/4627384 PMC5749293

[38] Puga Yung GL, Wakley T, Kouklas A, Seebach JD. Dendritic cells in xenotransplantation: shaping the cellular immune response toward tolerance. Xenotransplantation. 2025;32:e70037. 40243284 10.1111/xen.70037 PMC12005074

[39] Lu TY, Xu XL, Du XG, Wei JH, Yu JN, Deng SL, et al. Advances in innate immunity to overcome immune rejection during xenotransplantation. Cells. 2022;11:3865. 36497122 10.3390/cells11233865 PMC9735653

[40] Al-Mohanna F, Saleh S, Parhar RS, Khabar K, Collison K. Human neutrophil gene expression profiling following xenogeneic encounter with porcine aortic endothelial cells: the occult role of neutrophils in xenograft rejection revealed. J Leukoc Biol. 2005;78:51–61. 15809289 10.1189/jlb.0904494

[41] Ide K, Wang H, Tahara H, Liu J, Wang X, Asahara T, et al. Role for CD47-SIRPalpha signaling in xenograft rejection by macrophages. Proc Natl Acad Sci U S A. 2007;104:5062–6. 17360380 10.1073/pnas.0609661104 PMC1829264

[42] Reemtsma K, McCracken BH, Schlegel JU, Pearl MA, Pearce CW, Dewitt CW, et al. Renal heterotransplantation in man. Ann Surg. 1964;160:384–410. 14206847 10.1097/00000658-196409000-00006 PMC1408776

[43] Vogel CW, Müller-Eberhard HJ. Cobra venom factor: improved method for purification and biochemical characterization. J Immunol Methods. 1984;73:203–20. 6491300 10.1016/0022-1759(84)90045-0

[44] Miyagawa S, Shirakura R, Matsumiya G, Fukushima N, Nakata S, Matsuda H, et al. Prolonging discordant xenograft survival with anticomplement reagents K76COOH and FUT175. Transplantation. 1993;55:709–13. 8475539 10.1097/00007890-199304000-00004

[45] Weisman HF, Bartow T, Leppo MK, Marsh HC Jr, Carson GR, Concino MF, et al. Soluble human complement receptor type 1: in vivo inhibitor of complement suppressing post-ischemic myocardial inflammation and necrosis. Science. 1990;249:146–51. 2371562 10.1126/science.2371562

[46] Souza DG, Esser D, Bradford R, Vieira AT, Teixeira MM. APT070 (Mirococept), a membrane-localised complement inhibitor, inhibits inflammatory responses that follow intestinal ischaemia and reperfusion injury. Br J Pharmacol. 2005;145:1027–34. 15951831 10.1038/sj.bjp.0706286 PMC1576234

[47] Waytes AT, Rosen FS, Frank MM. Treatment of hereditary angioedema with a vapor-heated C1 inhibitor concentrate. N Engl J Med. 1996;334:1630–4. 8628358 10.1056/NEJM199606203342503

[48] Thomas TC, Rollins SA, Rother RP, Giannoni MA, Hartman SL, Elliott EA, et al. Inhibition of complement activity by humanized anti-C5 antibody and single-chain. Fv Mol Immunol. 1996;33:1389–401. 9171898 10.1016/s0161-5890(96)00078-8

[49] Locke JE, Magro CM, Singer AL, Segev DL, Haas M, Hillel AT, et al. The use of antibody to complement protein C5 for salvage treatment of severe antibody-mediated rejection. Am J Transpl. 2009;9:231–5. 10.1111/j.1600-6143.2008.02451.x 18976298

[50] Higgins PJ, Ko JL, Lobell R, Sardonini C, Alessi MK, Yeh CG. A soluble chimeric complement inhibitory protein that possesses both decay-accelerating and factor I cofactor activities. J Immunol. 1997;158:2872–81. 9058824

[51] Morikis D, Assa-Munt N, Sahu A, Lambris JD. Solution structure of Compstatin, a potent complement inhibitor. Protein Sci. 1998;7:619–27. 9541394 10.1002/pro.5560070311 PMC2143948

[52] Okada N, Asai S, Hotta A, Miura N, Ohno N, Farkas I, et al. Increased inhibitory capacity of an anti-C5a complementary peptide following acetylation of N-terminal alanine. Microbiol Immunol. 2007;51:439–43. 17446684 10.1111/j.1348-0421.2007.tb03918.x

[53] Röth A, Nishimura JI, Nagy Z, Gaàl-Weisinger J, Panse J, Yoon SS, et al. The complement C5 inhibitor crovalimab in paroxysmal nocturnal hemoglobinuria. Blood. 2020;135:912–20. 31978221 10.1182/blood.2019003399 PMC7082616

[54] Mastellos DC, Blom AM, Connolly ES, Daha MR, Geisbrecht BV, Ghebrehiwet B, et al. “Stealth” corporate innovation: an emerging threat for therapeutic drug development. Nat Immunol. 2019;20:1409–13. 31562490 10.1038/s41590-019-0503-1 PMC7368001

[55] Risitano AM, Röth A, Soret J, Frieri C, de Fontbrune FS, Marano L et al. Addition of iptacopan, an oral factor B inhibitor, to eculizumab in patients with paroxysmal nocturnal haemoglobinuria and active haemolysis: an open-label, single-arm, phase 2, proof-of-concept trial. Lancet Haematol. 202;8:e344-e354. 10.1016/S2352-3026(21)00028-4 33765419

[56] Caravaca-Fontán F, Gutiérrez E, Sevillano ÁM, Praga M. Targeting complement in IgA nephropathy. Clin Kidney J. 2023;16(Suppl 2):ii28–39. 38053977 10.1093/ckj/sfad198 PMC10695513

[57] Katopodis AG, Warner RG, Duthaler RO, Streiff MB, Bruelisauer A, Kretz O, et al. Removal of anti-Galalpha1,3Gal xenoantibodies with an injectable polymer. J Clin Invest. 2002;110:1869–77. 12488437 10.1172/JCI200216526 PMC151655

[58] Diamond LE, Byrne GW, Schwarz A, Davis TA, Adams DH, Logan JS. Analysis of the control of the anti-gal immune response in a non-human primate by galactose alpha1-3 galactose trisaccharide-polyethylene glycol conjugate. Transplantation. 2002;73:1780–7. 12085001 10.1097/00007890-200206150-00014

[59] Iwase H, Liu H, Li T, Zhang Z, Gao B, Hara H, et al. Therapeutic regulation of systemic inflammation in xenograft recipients. Xenotransplantation. 2017;24:10.1111/xen.12296. 10.1111/xen.12296 PMC5397335 28294424

[60] Piguet PF, Grau GE, Allet B, Vassalli P. Tumor necrosis factor/cachectin is an effector of skin and gut lesions of the acute phase of graft-vs.-host disease. J Exp Med. 1987;166:1280–9. 3316469 10.1084/jem.166.5.1280 PMC2189667

[61] Marquet RL, Bonthuis F, van Iijken M, Bouwman E, Wolvekamp MC, van Rooijen N, et al. Primary nonfunction of islet xenografts: the role of macrophages. Transpl Int. 1994;7(Suppl 1):S660-2. 11271333 10.1111/j.1432-2277.1994.tb01467.x

[62] Lo PC, Maeda A, Kodama T, Takakura C, Yoneyama T, Sakai R, et al. The novel immunosuppressant prenylated quinolinecarboxylic acid-18 (PQA-18) suppresses macrophage differentiation and cytotoxicity in xenotransplantation. Immunobiology. 2019;224:575–84. 30967296 10.1016/j.imbio.2019.04.003

[63] Hayashi S, Leu D, Yamii Y, Mei G, Takagi H, Nakao A. Effect of adenovirus-mediated transfer of the CTLA4IG gene in hamster-to-rat xenotransplantation. Transplantation. 2005;80:494–9. 16123724 10.1097/01.tp.0000168151.83816.f4

[64] Ferrant JL, Benjamin CD, Cutler AH, Kalled SL, Hsu YM, Garber EA, et al. The contribution of Fc effector mechanisms in the efficacy of anti-CD154 immunotherapy depends on the nature of the immune challenge. Int Immunol. 2004;16:1583–94. 15466914 10.1093/intimm/dxh162

[65] Miura S, Habibabady ZA, Pollok F, Ma M, Rosales IA, Kinoshita K, et al. TNX-1500, a crystallizable fragment-modified anti-CD154 antibody, prolongs nonhuman primate cardiac allograft survival. Am J Transplant. 2023;23:1182–93. 37030662 10.1016/j.ajt.2023.03.025 PMC10524282

[66] Anwar IJ, Berman DM, DeLaura I, Gao Q, Willman MA, Miller A, et al. The anti-CD40L monoclonal antibody AT-1501 promotes islet and kidney allograft survival and function in nonhuman primates. Sci Transl Med. 2023;15:eadf6376. 37647390 10.1126/scitranslmed.adf6376 PMC10990482

[67] Benda B, Ljunggren HG, Peach R, Sandberg JO, Korsgren O. Co-stimulatory molecules in islet xenotransplantation: CTLA4Ig treatment in CD40 ligand-deficient mice. Cell Transpl. 2002;11:715–20. 10.3727/000000002783985440 12518898

[68] Alwayn IP, Xu Y, Basker M, Wu C, Buhler L, Lambrigts D. Effects of specific anti-B and/or anti-plasma cell immunotherapy on antibody production in baboons: depletion of CD20- and CD22-positive B cells does not result in significantly decreased production of anti-alphaGal antibody. Xenotransplantation. 2001;8:157–71. 11472623 10.1034/j.1399-3089.2001.008003157.x

[69] Meier RPH, Pierson RN 3rd, Fishman JA, Buhler LH, Bottino R, Ladowski JM, et al. International xenotransplantation association (IXA) position paper on kidney xenotransplantation. Xenotransplantation. 2025;32:e70003. 40198240 10.1111/xen.70003

[70] Kawai T, Williams WW, Elias N, Fishman JA, Crisalli K, Longchamp A, et al. Xenotransplantation of a porcine kidney for end-stage kidney disease. N Engl J Med. 2025;392:1933–40. 39927618 10.1056/NEJMoa2412747

[71] Inverardi L, Clissi B, Stolzer AL, Bender JR, Sandrin MS, Pardi R. Human natural killer lymphocytes directly recognize evolutionarily conserved oligosaccharide ligands expressed by xenogeneic tissues. Transplantation. 1997;63:1318–30. 9158028 10.1097/00007890-199705150-00021

[72] Miyagawa S, Nakai R, Yamada M, Tanemura M, Ikeda Y, Taniguchi N, et al. Regulation of natural killer cell-mediated swine endothelial cell lysis through genetic remodeling of a glycoantigen. J Biochem. 1999;126:1067–73. 10578058 10.1093/oxfordjournals.jbchem.a022551

[73] Artrip JH, Kwiatkowski P, Michler RE, Wang SF, Tugulea S, Ankersmit J, et al. Target cell susceptibility to lysis by human natural killer cells is augmented by alpha(1,3)-galactosyltransferase and reduced by alpha(1, 2)-fucosyltransferase. J Biol Chem. 1999;274:10717–22. 10196142 10.1074/jbc.274.16.10717

[74] Baumann BC, Schneider MK, Lilienfeld BG, Antsiferova MA, Rhyner DM, Hawley RJ, et al. Endothelial cells derived from pigs lacking Gal alpha(1,3)Gal: no reduction of human leukocyte adhesion and natural killer cell cytotoxicity. Transplantation. 2005;79:1067–72. 15880045 10.1097/01.tp.0000157231.11083.7c

[75] Christiansen D, Mouhtouris E, Milland J, Zingoni A, Santoni A, Sandrin MS. Recognition of a carbohydrate xenoepitope by human NKRP1A (CD161). Xenotransplantation. 2006;13:440–6. 16925668 10.1111/j.1399-3089.2006.00332.x

[76] Miyagawa S, Kubo T, Matsunami K, Kusama T, Beppu K, Nozaki H, et al. Delta-short consensus repeat 4-decay accelerating factor (DAF: CD55) inhibits complement-mediated cytolysis but not NK cell-mediated cytolysis. J Immunol. 2004;173:3945–52. 15356143 10.4049/jimmunol.173.6.3945

[77] Sasaki H, Xu XC, Mohanakumar T. HLA-E and HLA-G expression on porcine endothelial cells inhibit xenoreactive human NK cells through CD94/NKG2-dependent and -independent pathways. J Immunol. 1999;163:6301–5. 10570324

[78] Matsunami K, Miyagawa S, Nakai R, Murase A, Shirakura R. The possible use of HLA-G1 and G3 in the inhibition of NK cell-mediated swine endothelial cell lysis. Clin Exp Immunol. 2001;126:165–72. 11678914 10.1046/j.1365-2249.2001.01622.x PMC1906174

[79] Matsunami K, Miyagawa S, Nakai R, Yamada M, Shirakura R. Modulation of the leader peptide sequence of the HLA-E gene up-regulates its expression and down-regulates natural killer cell-mediated swine endothelial cell lysis. Transplantation. 2002;73:1582–9. 12042643 10.1097/00007890-200205270-00010

[80] Matsunami K, Kusama T, Okura E, Shirakura R, Fukuzawa M, Miyagawa S. Involvement of position-147 for HLA-E expression. Biochem Biophys Res Commun. 2006;347:692–7. 16844086 10.1016/j.bbrc.2006.06.146

[81] Maeda A, Kawamura T, Ueno T, Usui N, Eguchi H, Miyagawa S. The suppression of inflammatory macrophage-mediated cytotoxicity and proinflammatory cytokine production by transgenic expression of HLA-E. Transpl Immunol. 2013;29:76–81. 23994719 10.1016/j.trim.2013.08.001

[82] Esquivel EL, Maeda A, Eguchi H, Asada M, Sugiyama M, Manabe C, et al. Suppression of human macrophage-mediated cytotoxicity by transgenic swine endothelial cell expression of HLA-G. Transpl Immunol. 2015;32:109–15. 25559170 10.1016/j.trim.2014.12.004

[83] Maeda A, Kogata S, Toyama C, Lo PC, Okamatsu C, Yamamoto R, et al. The innate cellular immune response in xenotransplantation. Front Immunol. 2022;13:858604. 35418992 10.3389/fimmu.2022.858604 PMC8995651

[84] Miyagawa S, Maeda A, Toyama C, Kogata S, Okamatsu C, Yamamoto R, et al. Aspects of the complement system in new era of xenotransplantation. Front Immunol. 2022;13:860165. 35493484 10.3389/fimmu.2022.860165 PMC9046582

[85] Matsumoto M, Tanaka T, Kaisho T, Sanjo H, Copeland NG, Gilbert DJ, et al. A novel LPS-inducible C-type lectin is a transcriptional target of NF-IL6 in macrophages. J Immunol. 1999;163:5039–48. 10528209

[86] Meyers JH, Sabatos CA, Chakravarti S, Kuchroo VK. The TIM gene family regulates autoimmune and allergic diseases. Trends Mol Med. 2005;11:362–9. 16002337 10.1016/j.molmed.2005.06.008

[87] Hanayama R, Miyasaka K, Nakaya M, Nagata S. MFG-E8-dependent clearance of apoptotic cells, and autoimmunity caused by its failure. Curr Dir Autoimmun. 2006;9:162–72. 16394660 10.1159/000090780

[88] Al-Mohanna F, Saleh S, Parhar RS, Khabar K, Collison K. Human neutrophil gene expression profiling following xenogeneic encounter with porcine aortic endothelial cells: the occult role of neutrophils in xenograft rejection revealed. J Leukoc Biol. 2005;78:51–61. 15809289 10.1189/jlb.0904494

[89] Schofield ZV, Woodruff TM, Halai M, Wu MC, Cooper MA. Neutrophils—a key component of ischemia-reperfusion injury. Shock. 2013;40:463–70. 24088997 10.1097/SHK.0000000000000044

[90] Saleh SM, Parhar RS, Al-Hejailan RS, Bakheet RH, Khaleel HS, Khalak HG, et al. Idtification of the tetraspanin CD82 as a new barrier to xenotransplantation. J Immunol. 2013;191:2796–805. 23872050 10.4049/jimmunol.1300601 PMC3748337

[91] Takase K, Gadomska K, Maeda A, Matsu J, Nakahata K, Nomura M, et al. HLA-Class Ib Expression Suppresses Neutrophil Xenogeneic Immune Responses Against Pig Cells. J Clin Exp Nephrol. 2025;10:292.

[92] Deuse T, Hu X, Agbor-Enoh S, Jang MK, Alawi M, Saygi C, et al. The SIRPα-CD47 immune checkpoint in NK cells. J Exp Med. 2021;218:e20200839. 33416832 10.1084/jem.20200839 PMC7802363

[93] Oldenborg PA, Zheleznyak A, Fang YF, Lagenaur CF, Gresham HD, Lindberg FP. Role of CD47 as a marker of self on red blood cells. Science. 2000;288:2051–4. 10856220 10.1126/science.288.5473.2051

[94] Hayat SMG, Bianconi V, Pirro M, Jaafari MR, Hatamipour M, Sahebkar A. CD47: role in the immune system and application to cancer therapy. Cell Oncol (Dordr). 2020;43:19–30. 31485984 10.1007/s13402-019-00469-5 PMC12990683

[95] Chen M, Wang Y, Wang H, Sun L, Fu Y, Yang YG. Elimination of donor CD47 protects against vascularized allograft rejection in mice. Xenotransplantation. 2019;26:e12459. 30136356 10.1111/xen.12459 PMC6387643

[96] Takeuchi K, Ariyoshi Y, Shimizu A, Okumura Y, Cara-Fuentes G, Garcia GE, et al. Expression of human CD47 in pig glomeruli prevents proteinuria and prolongs graft survival following pig-to-baboon xenotransplantation. Xenotransplantation. 2021;28:e12708. 34418164 10.1111/xen.12708 PMC8957703

[97] Nomura S, Ariyoshi Y, Watanabe H, Pomposelli T, Takeuchi K, Garcia G, et al. Transgenic expression of human CD47 reduces phagocytosis of porcine endothelial cells and podocytes by baboon and human macrophages. Xenotransplantation. 2020;27:e12549. 31495971 10.1111/xen.12549 PMC7007337

[98] Thakar CV, Zahedi K, Revelo MP, Wang Z, Burnham CE, Barone S, et al. Identification of thrombospondin 1 (TSP-1) as a novel mediator of cell injury in kidney ischemia. J Clin Invest. 2005;115:3451–9. 16294224 10.1172/JCI25461 PMC1283940

[99] Lario S, Bescós M, Campos B, Mur C, Luque P, Alvarez R, et al. Thrombospondin-1 mRNA expression in experimental kidney transplantation with heart-beating and non-heart-beating donors. J Nephrol. 2007;20:588–95. 17918145

[100] Gardai SJ, Xiao YQ, Dickinson M, Nick JA, Voelker DR, Greene KE, et al. By binding SIRPalpha or calreticulin/CD91, lung collectins act as dual function surveillance molecules to suppress or enhance inflammation. Cell. 2003;115:13–23. 14531999 10.1016/s0092-8674(03)00758-x

[101] Janssen WJ, McPhillips KA, Dickinson MG, Linderman DJ, Morimoto K, Xiao YQ, et al. Surfactant proteins A and D suppress alveolar macrophage phagocytosis via interaction with SIRP alpha. Am J Respir Crit Care Med. 2008;178:158–67. 18420961 10.1164/rccm.200711-1661OC PMC2453510

[102] Jiaravuthisan P, Maeda A, Takakura C, Wang HT, Sakai R, Mohd Shabri A, et al. A membrane-type surfactant protein D (SP-D) suppresses macrophage-mediated cytotoxicity in swine endothelial cells. Transpl Immunol. 2018;47:44–8. 29425774 10.1016/j.trim.2018.02.003

[103] Toyama C, Maeda A, Kogata S, Yamamoto R, Masahata K, Ueno T, et al. Suppression of xenogeneic innate immune response by a membrane-type human surfactant protein-A. Exp Ther Med. 2022;24:590. 35949334 10.3892/etm.2022.11527 PMC9353545

[104] Ohtani K, Suzuki Y, Wakamiya N. Biological functions of the novel collectins CL-L1, CL-K1, and CL-P1. J Biomed Biotechnol. 2012;2012:493945. 22570530 10.1155/2012/493945 PMC3336186

[105] Fournier B, Andargachew R, Robin AZ, Laur O, Voelker DR, Lee WY, et al. Surfactant protein D (Sp-D) binds to membrane-proximal domain (D3) of signal regulatory protein alpha (SIRPalpha), a site distant from binding domain of CD47, while also binding to analogous region on signal regulatory protein beta (SIRPbeta). J Biol Chem. 2012;287:19386–98. 22511785 10.1074/jbc.M111.324533 PMC3365977

[106] Shi L, Bian Z, Kidder K, Liang H, Liu Y. Non-lyn Src family kinases activate SIRPα–SHP-1 to inhibit PI3K–Akt2 and dampen proinflammatory macrophage polarization. J Immunol. 2021;207:1419–27. 34348974 10.4049/jimmunol.2100266 PMC8387419

[107] Sakai R, Maeda A, Choi TV, Lo PC, Jiaravuthiasan P, Shabri AM, et al. Human CD200 suppresses macrophage-mediated xenogeneic cytotoxicity and phagocytosis. Surg Today. 2018;48:119–26. 28573328 10.1007/s00595-017-1546-2

[108] Noguchi Y, Maeda A, Lo PC, Takakura C, Haneda T, Kodama T, et al. Human TIGIT on porcine aortic endothelial cells suppresses xenogeneic macrophage-mediated cytotoxicity. Immunobiology. 2019;224:605–13. 31402149 10.1016/j.imbio.2019.07.008

[109] Maeda A, Kawamura T, Ueno T, Usui N, Miyagawa S. Monocytic suppressor cells derived from human peripheral blood suppress xenogenic immune reaction. Xenotransplantation. 2014;21:46–56. 24372857 10.1111/xen.12067

[110] Maeda A, Kawamura T, Nakahata K, Ueno T, Usui N, Hiroshi H, et al. Regulation of macrophage-mediated xenocytotoxicity by overexpression of alpha 2,6-sialyltransferase in swine endothelial cells. Transplant Proc. 2014;46:1256–8. 24815175 10.1016/j.transproceed.2013.11.026

[111] Maeda A, Eguchi H, Nakahta K, Lo PC, Yamanaka K, Kawamura T, et al. Monocytic MDSCs regulate macrophage-mediated xenogenic cytotoxicity. Transpl Immunol. 2015;33:140–5. 26209355 10.1016/j.trim.2015.07.002

[112] Iemitsu K, Sakai R, Maeda A, Gadomska K, Kogata S, Yasufuku D, et al. The hybrid CL-SP-D molecule has the potential to regulate xenogeneic rejection by human neutrophils more efficiently than CD47. Transpl Immunol. 2024;84:102020. 38452982 10.1016/j.trim.2024.102020

[113] Newton JP, Buckley CD, Jones EY, Simmons DL. Residues on both faces of the first immunoglobulin fold contribute to homophilic binding sites of PECAM-1/CD31. J Biol Chem. 1997;272:20555–63. 9252369 10.1074/jbc.272.33.20555

[114] Lertkiatmongkol P, Liao D, Mei H, Hu Y, Newman PJ. Endothelial functions of platelet/endothelial cell adhesion molecule-1(CD31). Curr Opin Hematol. 2016;23:253–9. 27055047 10.1097/MOH.0000000000000239 PMC4986701

[115] Sachs UJH, Andrei-Selmer CL, Maniar A, Weiss T, Paddock C, Orlova VV, et al. The neutrophil-specific antigen CD177 is a counter-receptor for platelet endothelial cell adhesion molecule-1 (CD31). J Biol Chem. 2007;282:23603–12. 17580308 10.1074/jbc.M701120200

[116] Noguchi Y, Maeda A, Wang HT, Takakura C, Lo PC, Kodama T, et al. Human CD31 on Swine endothelial cells induces SHP-1 phosphorylation in macrophages. Transplant Proc. 2020;52:1913–5. 32402461 10.1016/j.transproceed.2020.01.140

[117] Kogata S, Lo PC, Maeda A, Okamatsu C, Sato K, Yamamoto R, et al. Suppression of macrophage-mediated xenogeneic rejection by the ectopic expression of human CD177. Transpl Immunol. 2022;74:101663. 35835297 10.1016/j.trim.2022.101663

[118] Wang HT, Maeda A, Sakai R, Lo PC, Takakura C, Jiaravuthisan P, et al. Human CD31 on porcine cells suppress xenogeneic neutrophil-mediated cytotoxicity via the inhibition of NETosis. Xenotransplantation. 2018;25:e12396. 29635708 10.1111/xen.12396

[119] Yoneyama T, Maeda A, Kogata S, Toyama C, Lo PC, Masahata K, et al. The regulation of neutrophil extracellular trap-induced tissue damage by human CD177. Transplant Direct. 2021;7:e734. 34549086 10.1097/TXD.0000000000001175 PMC8439991

[120] Miyagawa S, Maeda A, Matsunami K, Eguchi H, Okuyama H. Aiming for the clinical application of xenotransplantation. In: Heart and lung transplantation: historical developments, clinical achievements, and future perspectives. Springer Singapore; 2025. p. 277–89.

